# Prospective associations between breast feeding, metabolic health, inflammation and bone density in women with prior gestational diabetes mellitus

**DOI:** 10.1136/bmjdrc-2024-004117

**Published:** 2024-05-21

**Authors:** Ines Hebeisen, Elena Gonzalez Rodriguez, Amar Arhab, Justine Gross, Sybille Schenk, Leah Gilbert, Katrien Benhalima, Antje Horsch, Dan Yedu Quansah, Jardena J Puder

**Affiliations:** 1 Obstetric Service, Department Woman-Mother-Child, Lausanne University Hospital, Lausanne, Switzerland; 2 University of Lausanne, Lausanne, Switzerland; 3 Interdisciplinary Center of Bone Diseases, Bone and Joint Department, Lausanne University Hospital, Lausanne, Switzerland; 4 Service of Endocrinology, Diabetes and Metabolism, Department of Medicine, CHUV, Lausanne, Switzerland; 5 Service of Obsterics, Department Woman-Mother-Child, Lausanne University Hospital, Lausanne, Switzerland; 6 Department of Endocrinology, KUL UZ Gasthuisberg, Leuven, Belgium; 7 Neonatology service, Department Woman-Mother-Child, Lausanne University Hospital, Lausanne, Switzerland; 8 Institute of Higher Education and Research in Healthcare (IUFRS), University of Lausanne, Lausanne, Switzerland

**Keywords:** GDM, Body Fat, Bone Density, Body Weight

## Abstract

**Introduction:**

The aim of the study is to investigate prospective associations between breastfeeding and metabolic outcomes, inflammation, and bone density in women with prior gestational diabetes mellitus (GDM).

**Research design and methods:**

We prospectively included 171 women with GDM from the MySweetheart trial. Women were followed during pregnancy (from 24 up to 32 weeks’ gestational age) up to 1 year postpartum. Outcomes included weight, weight retention, body composition, insulin resistance and secretion indices, C reactive protein (CRP), and bone density. We compared differences in the associations between breastfeeding and health outcomes between women who breast fed <6 months vs ≥6 months. Analyses were adjusted for potential medical and sociodemographic confounders.

**Results:**

Breastfeeding initiation was 94.2% (n=161) and mean breastfeeding duration was 6.6 months. Breastfeeding duration was independently associated with lower weight, weight retention, body fat, visceral adipose tissue, lean mass, CRP, insulin resistance (Homeostatic Model Assessment for Insulin Resistance), and insulin secretion (Homeostatic Model Assessment of β-cell index) at 1 year postpartum (all p≤0.04) after adjusting for confounders. Breastfeeding was associated with higher insulin resistance-adjusted insulin secretion (Insulin Secretion-Sensitivity Index-2) in the unadjusted analyses only. There was no association between breastfeeding duration and bone density. Compared with <6 months, breastfeeding duration ≥6 months was associated with lower weight, weight retention, body fat, fat-free mass as well as lower CRP at 1 year postpartum (all p<0.05) after adjusting for confounders.

**Conclusions:**

Longer breastfeeding duration among women with prior GDM was associated with lower insulin resistance, weight, weight retention, body fat and inflammation, but not lower bone density at 1 year postpartum. Breastfeeding for ≥6 months after GDM can help to improve cardiometabolic health outcomes 1 year after delivery.

**WHAT IS ALREADY KNOWN ON THIS TOPIC**

- Breastfeeding is a modifiable risk factor for cardiometabolic diseases; however, there is no consensus on a recommended breastfeeding duration to improve maternal health.
- There are limited data on the relationship between breastfeeding and body composition, insulin resistance and secretion, inflammation, and bone density in women with gestational diabetes mellitus (GDM).

**WHAT THIS STUDY ADDS**

- We found that breastfeeding duration was associated with lower weight, weight retention, body fat, lean mass, visceral adipose tissue, insulin resistance, insulin secretion and C reactive protein, but not insulin resistance-adjusted insulin secretion, nor bone mineral density at 1 year postpartum in women after GDM.
- Breastfeeding for more than 6 months improved cardiometabolic health in women after GDM.

**HOW THIS STUDY MIGHT AFFECT RESEARCH, PRACTICE OR POLICY**

- Our study is the first to show that breastfeeding for more than 6 months is protective of adverse metabolic outcomes without compromising bone health in women with prior GDM. Future studies can expand on our findings to investigate the effect of breastfeeding on bone density in women with prior GDM. In practice, breastfeeding for more than 6 months, especially for women with GDM, should be encouraged by healthcare professionals.

## Introduction

Women with gestational diabetes mellitus (GDM) have a more than sixfold increased risk of type 2 diabetes mellitus (T2DM)^1^ and a twofold higher risk of cardiovascular diseases (CVDs).^2^ Therefore, improving cardiometabolic health in this young, high-risk group is crucial.

Breastfeeding is a modifiable protective factor for cardiometabolic diseases and is associated with a lower future risk of glucose intolerance, T2DM, metabolic syndrome, and CVD, independent of known confounders.^3–5^ Breastfeeding can also contribute to loss of weight and body fat.^6^ In women with GDM, breastfeeding has been associated with lower postpartum body mass index (BMI) and improved insulin resistance.^7^ However, there is lack of data on the impact of breast feeding on body fat, lean mass or bone mineral density (BMD) in this population. There are conflicting data regarding its effect on body composition and BMD in the general population.^8–13^ While an intervention to promote breastfeeding duration did not result in lower percentage of body fat in a large cluster-randomized trial,^14^ one cohort study showed a beneficial association between breastfeeding and fat mass index (FMI) in women having had a child in the last 5 years^9^ and two prospective studies suggested that exclusive breast feeding was associated with lower fat mass than mixed breastfeeding.^15 16^ In addition, breastfeeding has been associated with a lower visceral adipose tissue in some studies.^17–19^ Although existing studies that investigated the relationship between breastfeeding duration and lean mass are limited, no significant relationships have been reported.^10 11^

Although some studies have associated breastfeeding with a reduction in visceral adipose tissue (VAT),^17 18^ this has not been shown in women with prior GDM.^20 21^ Regarding insulin secretion (assessed by the disposition index), the impact of breastfeeding after GDM has shown a beneficial association in some,^21 22^ but not all studies.^23 24^ Similar inconsistency is observed regarding the association between breastfeeding and inflammatory parameters,^20 23 25 26^ which tend to be higher in women with GDM compared with controls.^27^ Studying the impact of breastfeeding on metabolic health parameters and inflammation in this population is important, since the underlying mechanism through which breastfeeding mitigates cardiometabolic risk remains unclear. Emerging research indicates that metabolic syndrome and inflammation are associated with decreased BMD.^28 29^ Women with prior GDM have lower BMD^30^ compared with healthy controls, as well as an increased risk of hip and other fractures later in life.^31^ Additionally, breastfeeding is associated with a reduction in BMD, that may be transitory.^13^ To date, there have been no studies on the impact of breastfeeding on BMD in women with prior GDM.

Existing studies in women with GDM are mostly retrospective, and rarely account for metabolic risk or protective factors prior to conception, lifestyle factors or adverse pregnancy outcomes that might influence breastfeeding initiation and duration.^32^ Furthermore, there is no consensus on the recommended breastfeeding duration for improved maternal health, as the recommendation for 6-month exclusive breastfeeding by the WHO^33^ and other international health organizations^34^ is based on infant health. Therefore, and to investigate the effect of breastfeeding on overall health, we aimed to investigate the prospective associations between breastfeeding and maternal metabolic health, inflammation, and bone density at 1 year postpartum in a multiethnic cohort of women with prior GDM. We specifically evaluated weight, weight retention, body fat, fat-free mass, VAT, insulin resistance, and insulin secretion. We also tested if all these health outcomes differed between women who breastfed for ≥6 months vs <6 months.

## Subjects, materials and methods

### Study design and participants

This prospective study is a secondary analysis of the ‘MySweetheart trial’ (NCT02890693), which tested the effect of an interdisciplinary prepartum and postpartum lifestyle and psychosocial intervention in women with GDM. The study protocol has been previously described.^35^ Of the 211 women included in the trial (n=105 in usual care, n=106 in the intervention group), the current analysis excluded women who had missing data on breastfeeding duration (n=40). All 171 women included completed the postpartum visit of 6–8 weeks and n=165 completed the 1-year postpartum follow-up.

### GDM management and patient follow-up

Participants in the usual care group were followed according to the current guidelines of the American Diabetes Association and the Endocrine Society guidelines.^36 37^ Women were first seen at 24–32 weeks of GA (gestational age) by a physician or diabetes specialist nurse, who then followed them until delivery. They received information on GDM, dietary advice by a registered dietician and tailored recommendations regarding lifestyle changes and gestational weight gain (GWG).^38^ Glucose-lowering medication (insulin and/or very rarely metformin) was introduced if capillary glucose values remained above target (fasting glucose >5.3 mmol/L, 1-hour postprandial glucose >8 mmol/L or 2-hour postprandial glucose >7 mmol/L) despite lifestyle modifications. All women were counseled on the benefits of breastfeeding. At 6–8 weeks and 1 year postpartum, women underwent a 75 g oral glucose tolerance test (oGTT) and general advice on lifestyle counseling was provided, but no medication prescribed.

On top of the usual care, women in the intervention group had a total of nine individual interdisciplinary lifestyle sessions in the perinatal period, two workshops and a coach support mostly through telemedicine. It focused on tailored behavioral and psychosocial strategies to improve diet, physical activity, mental health, social support, and encouraged breastfeeding.^6^

### Measures

Breastfeeding duration served as the predictor, and we assessed metabolic, inflammatory, and bone density measures at 1 year postpartum.

#### Sociodemographic, descriptive health, and lifestyle characteristics

Sociodemographic variables including age, educational level, and ethnicity were assessed during the first GDM visit (24–32 GA). Data on medical characteristics including pre-pregnancy weight, parity, gravida, GDM in previous pregnancy, family history of diabetes, and medical treatment were extracted from the patients’ medical chart or assessed during a structured face-to-face interview.

Dietary intake was assessed with a validated Food Frequency Questionnaire (FFQ), developed for adults who live in the French part of Switzerland. Details of this FFQ are described elsewhere.^39^ Physical activity (PA) was assessed with the GENEActiv accelerometer worn for 10 consecutive days on the right wrist.^40^ Both the FFQ and the PA were assessed during pregnancy and at 1 year postpartum.

#### Pregnancy outcomes

GA at delivery, and adverse pregnancy outcomes including gestational hypertension, pre-eclampsia, placenta previa, cesarean section, prematurity, small and large for gestational age,^41^ intrauterine growth restriction, and hospitalization in neonatology were extracted from the patients’ medical chart.

#### Breastfeeding duration

We assessed breastfeeding duration in months both at 6–8 weeks and at 1 year postpartum using a self-report questionnaire (without differentiating between exclusive and non-exclusive breastfeeding). If patients discontinued within the first month, we recorded a duration of 0.5 months, representing the median value between 0 and 1 month. When breastfeeding information was missing from the questionnaires (n=8), we relied on the information collected at the postpartum clinical visits at which women were asked if they were still breastfeeding, and if not, when they had stopped.

#### Metabolic health outcomes, inflammatory markers, and bone density

##### Anthropometry and body composition

Pre‐pregnancy weight was extracted from participants’ medical charts or was rarely self‐reported at the first GDM visit if missing. We measured height and weight (Seca model 220, Hamburg, Germany) at the first GDM visit. Weight was also measured at the postpartum visits. Postpartum weight retention (PPWR) at 1 year was defined as the difference in pre-pregnancy weight and weight at 1 year postpartum.

Body fat and fat-free mass were estimated from reactance and resistance values according to Kyle’s equation,^42^ using bioelectrical impedance analysis (BIA, Akern BIA 101) at 6–8 weeks and 1 year postpartum. At the 1-year postpartum visit, body composition (fat and lean mass) was assessed by dual X-ray absorptiometry (DXA) using Lunar iDXA (GE Healthcare) in 109 women who signed an additional consent form for this procedure. VAT was determined using DXA CoreScan software, and FMI (kg/m^2^) was calculated as the ratio of fat mass (kg) to the square of height (m^2^).

We measured HbA1c with a chemical photometric method (conjugation with boronate‐Afinion) at the first GDM visit, and with a high‐performance liquid chromatography method in the postpartum according to international guidelines.^43^

##### Insulin resistance/secretion indices

Women underwent an oGTT at 1 year postpartum. We measured glucose and insulin values every 30 min over 2 hours to calculate insulin resistance and secretion indices. The Homeostatic Model Assessment for Insulin Resistance (HOMA-IR)^44^ was used as a measure of insulin sensitivity and Homeostatic Model Assessment of β-cell index (HOMA-B)^45^ as a measure of β-cell function. Whole body insulin sensitivity was estimated with the Matsuda index.^46^ Absolute insulin secretion was estimated using area under the curve (AUCins/glu).^46^ Insulin Secretion-Sensitivity Index-2 (ISSI-2), also known as disposition index, was expressed as the product of Matsuda index and AUCins/glu.^47^ Women were advised to avoid moving, smoking and breastfeeding during the oGTT to avoid influencing the results.^48^

##### Measurement of inflammation

We measured C reactive protein (CRP), tumor necrosis factor α (TNF-α), and interleukin 6 (IL-6) at 1 year postpartum. CRP was analyzed in serum aliquots using a latex-enhanced immunoturbidimetric assay on a Cobas 8000 autoanalyzer (Roche Diagnostics, Mannheim, Germany), with assay characteristics as reported by the manufacturer. TNF-α (U-PLEX Human TNF-α Antibody Set) and IL-6 (U-PLEX Human IL-6 Antibody Set) were measured using ELISA according to the manufacturer’s instructions.

#### Bone mineral density

BMD, and BMD T-score and Z-score of the vertebrae L1–L4 were assessed using Lunar iDXA. BMD was calculated in accordance with the International Society of Clinical Densitometry criteria.^49^

### Statistical analysis

All statistical analyses were performed with STATA/SE V.17.0 (StataCorp, Texas, USA). Sociodemographic and health characteristics were expressed as means (±SD) or number and percentages (%). Outcome variables were normally distributed, except for inflammatory markers (CRP, TNF-α and IL-6), for which natural log transformation was performed beforehand. Breastfeeding duration was considered a continuous variable (in months), or categorized into BF <6 vs BF ≥6 based on the median duration of breastfeeding in our sample (6 months) and in line with international recommendations to breastfeed for at least 6 months.^33 34^ Potential sociodemographic, lifestyle and health-related confounders were chosen based on previous literature^32 50–52^ and their potential relationship with our predictor (breastfeeding duration) and/or outcome variables (metabolic health, inflammation and bone density). We tested the following potential confounders: age, pre-pregnancy weight, pre-pregnancy BMI, weight at the first GDM visit, HbA1c at the first GDM visit, total caloric intake at first GDM visit, total PA at first GDM visit, time spent in moderate and vigorous PA at first GDM visit, ethnicity/nationality, educational level, parity, gravida, GDM in previous pregnancy, family history of diabetes, GWG throughout pregnancy, glucose-lowering treatment in pregnancy, pregnancy-induced hypertension, pre-eclampsia during pregnancy, placenta previa during pregnancy, cesarean section, prematurity, small for gestational age, intrauterine growth restriction, hospitalization in neonatology, food intake at 1 year postpartum, daily total PA at 1 year postpartum and time spent in moderate and vigorous physical activity at 1 year postpartum. Differences in potential confounders were assessed according to breastfeeding category (no or <6 months (BF <6) vs ≥6 months (BF ≥6) of breastfeeding), using an analysis of variance test for continuous variables or Χ^2^ test for categorical variables. Potential confounders that were significantly different between our two breastfeeding groups (pre-pregnancy BMI, educational level and glucose-lowering treatment during pregnancy) were included as confounders in our further analysis. Predictors and outcomes (body composition, metabolic health, inflammatory markers and bone density) at 1 year postpartum did not differ between the intervention and usual care groups. Mean breastfeeding duration (predictor) was 7 months in both groups. Similarly, the results and effect sizes of the relationship between breastfeeding duration and the different outcomes were similar when we restricted the analyses to the usual care group. Therefore, we pooled participants from both groups to increase the sample size and adjusted for group allocation in all analyses.

We performed partial correlations and multiple linear regression analysis to determine the relationship between breastfeeding duration and outcome variables, adjusting only for group allocation (model 1). In a second model, we also adjusted for the aforementioned confounders that were significantly different between BF <6 and BF ≥6 groups (pre-pregnancy BMI, educational level, and need for glucose-lowering treatment during pregnancy).

We also performed a multiple linear regression analysis to determine differences in health outcomes according to category (BF <6 vs BF ≥6) using the same two regression models. We did not correct for multiple testing due to the high correlations between several of the health outcomes, in particular the metabolic ones, and the known potential impact of breastfeeding on various health outcomes.^53^

In supplementary analyses, we investigated differences in adverse pregnancy outcomes as well as lifestyle variables at 1 year postpartum, according to breastfeeding category. For all statistical analyses, two-sided p<0.05 was considered to be statistically significant.

## Results

Mean age at the first GDM visit (n=171) was 33.5±5.8 years and mean pre-pregnancy weight was 68.6±14.7 kg (table 1).

**Table 1:** Sociodemographic and descriptive health characteristics of study participants according to breastfeeding category

	All (n=171)	BF <6 (n=69)	BF ≥6 (n=102)	
	**Mean±SD**	**Mean±SD**	**Mean±SD**	**P value**
Age (years)	33.5±5.8	32.6±6.7	34.1±4.6	0.08
Pre-pregnancy weight (kg)	68.6±14.7	72.5±16.6	66.3±12.7	0.01
Pre-pregnancy BMI (kg/m^2^)	25.5±5.1	26.7±5.7	24.7±4.5	0.01
Gestational weight gain throughout pregnancy (kg)	12.7±0.3	13.3±7.4	12.4±5.1	0.44
Weight at the first GDM visit (kg)	79.2±14.6	83.1±16.2	76.5±12.7	<0.01
HbA1c at the first GDM visit (%) (mmol/mol)	5.2±0.3 (33±3)	5.3±0.3 (34±3)	5.2±0.3 (33±3)	0.47
Total food intake at first GDM visit (kcal/day) (n=92)	1468.7±553.8	1549.5±527.6	1416.8±568.6	0.27
Total physical activity at first GDM visit (min/day) (n=149)	253.8±76.4	241.0±75.5	262.0±76.3	0.11
Time spent in moderate and vigorous physical activity at first GDM visit (min/day) (n=149)	108.0±46.6	105.3±53.0	109.6±42.3	0.59

	n (%)	n (%)	n (%)	
Ethnicity/nationality (n=157)				0.78
Switzerland	54 (34.4)	21 (33.3)	33 (35.1)	
Rest of Europe and North America	64 (40.8)	26 (41.3)	38 (40.4)	
Asia	13 (8.3)	6 (9.5)	7 (7.5)	
Africa	19 (12.1)	7 (11.1)	12 (12.8)	
Latin America	5 (3.2)	3 (4.8)	2 (2.1)	
Others	2 (1.3)	0 (0.00)	2 (2.1)	
Educational level (n=143)				0.01
Compulsory school incomplete*	2 (1.4)	0 (0.00)	2 (2.3)	
Compulsory school achieved	20 (14.0)	12 (21.8)	8 (9.1)	
High school	15 (10.5)	4 (7.3)	11 (12.5)	
General and vocational education	28 (19.6)	16 (29.1)	12 (13.6)	
University	78 (54.6)	23 (41.8)	55 (62.5)	
Parity (n=171)				0.42
0	99 (57.9)	40 (58.0)	59 (57.8)	
1	47 (27.5)	20 (29.0)	27 (26.5)	
2	12 (7.0)	2 (2.9)	10 (9.8)	
≥3	13 (7.6)	7 (10.1)	6 (5.9)	
Gravida (n=171)				0.44
1	72 (42.1)	28 (40.6)	44 (43.1)	
2	44 (25.7)	20 (29.0)	24 (23.5)	
≥3	5 (32.2)	21 (30.4)	34 (33.3)	
GDM in previous pregnancy (n=84)†				0.87
No	67 (79.8)	30 (79.0)	37 (80.4)	
Yes	17 (20.2)	8 (21.1)	9 (19.6)	
Family history of diabetes (n=169)‡				0.65
No	109 (65.1)	45 (67.2)	65 (63.7)	
Yes	59 (34.9)	22 (32.8)	37 (36.3)	
Glucose-lowering treatment in pregnancy (n=163)§				0.02
No	89 (54.6)	29 (43.9)	60 (61.9)	
Yes	74 (45.4)	37 (56.1)	37 (38.1)	

Data are expressed as n (%) for categorical variables or mean±SD for continuous variables.

*In Switzerland, compulsory schooling lasts 11 years. For statistical analysis, missing data were replaced by mean imputation.

†Only for women who had at least one previous pregnancy (n=99).

‡Family history of diabetes includes those with first-degree relationship of the participant (eg, mother, father, brother, sister, daughter, son).

§Treatment was insulin for all women, except in seven cases (4.1%) that were only treated with metformin and three cases (1.8%) that were treated with metformin and basal insulin.

BF <6, no or <6 months of breastfeeding; BF ≥6, ≥6 months of breastfeeding; BMI, body mass index; GDM, gestational diabetes mellitus.

Mean breastfeeding duration was 6.6 months, with a rate of breastfeeding initiation among the study participants of 94.2% (n=161). Specifically, 40.4% (n=69) of women either did not initiate (n=10) or breastfed for less than 6 months (BF <6), whereas 59.6% (n=102) breastfed for at least 6 months or longer (BF ≥6). The mean breastfeeding duration was 2.1 months in BF <6 women, and 9.7 months in BF ≥6 women.

BF <6 women had a significantly higher pre-pregnancy weight, pre-pregnancy BMI, and weight at first GDM visit compared with BF ≥6 women (all p≤0.01). In addition, they had a lower educational level and more frequently needed glucose-lowering treatment during pregnancy (all p≤0.02). There were no significant differences in adverse pregnancy outcomes such as pre-eclampsia, cesarean section, or neonatal hospitalization between BF <6 and BF ≥6 (online supplemental table 1), nor in dietary intake or PA at 1 year postpartum (online supplemental table 2).

10.1136/bmjdrc-2024-004117.supp1 Supplementary data

10.1136/bmjdrc-2024-004117.supp2 Supplementary data

### Associations between breastfeeding duration and metabolic health, inflammatory markers, and bone density

Breastfeeding duration inversely correlated with body composition at 1 year postpartum (table 2).

**Table 2:** Relationship between breastfeeding duration and metabolic health, C reactive protein (CRP), and bone density at 1 year postpartum in women with prior GDM

	Model 1*		Model 2†	
	**β (95% CI**)	**P value**	**β (95% CI**)	**P value**
Body composition				
Weight (kg)	−1.38 (−1.93, 0.84)	<0.001	−0.54 (−0.83, 0.24)	<0.001
Weight retention (kg)	−0.41 (−0.60, 0.22)	<0.001	−0.36 (−0.57, 0.15)	<0.01
Body fat (BIA, kg)	−0.98 (−1.33, 0.63)	<0.001	−0.42 (−0.61, 0.22)	<0.001
Fat-free mass (BIA, kg)	−0.54 (−0.75, 0.32)	<0.001	−0.26 (−0.42, 0.11)	<0.01
ΔBody composition between 1 year and 6–8 weeks post partum				
Weight difference (kg)	−0.31 (−0.47, 0.15)	<0.001	−0.21 (−0.38, 0.04)	0.01
Body fat difference (BIA, kg)	−0.28 (−0.42, 0.14)	<0.001	−0.19 (−0.33, 0.04)	0.01
Fat-free mass difference (BIA, kg)	−0.10 (−0.19, 0.02)	0.02	−0.09 (−0.18, 0.0004)	0.04
Body composition by DEXA				
Body mass (kg)	−0.94 (−1.42, 0.46)	<0.001	−0.43 (−0.68, 0.18)	<0.01
Lean mass (kg)	−0.38 (−0.64, 0.13)	<0.01	−0.22 (−0.42, 0.10)	0.04
Fat mass index (kg/m^2^)	−0.30 (−0.48, 0.12)	<0.01	−0.11 (−0.20, 0.03)	0.01
Visceral adipose tissue (kg)	−0.03 (−0.05, 0.01)	<0.01	−0.01 (−0.02, 0.001)	0.04
Insulin resistance/secretion				
HOMA-IR	−0.17 (−0.25, 0.10)	<0.001	−0.08 (−0.15, 0.02)	0.01
HOMA-B	−2.32 (−3.38, 1.25)	<0.001	−1.11 (−2.05, 0.17)	0.02
Matsuda	0.19 (0.06, 0.33)	<0.01	0.01 (−0.10, 0.13)	0.82
AUC	−0.02 (−0.03, 0.01)	<0.01	−0.01 (−0.02, 0.004)	0.20
ISSI-2	0.04 (0.01, 0.07)	0.01	0.02 (−0.02, 0.05)	0.30
Inflammation				
CRP (mg/L)*	−0.07 (−0.11, 0.03)	<0.01	−0.04 (−0.09, 0.003)	0.04
TNF-α (pg/mL)*	−0.01 (−0.04, 0.02)	0.55	−0.01 (−0.04, 0.02)	0.59
IL-6 (pg/mL)*	0.01 (−0.03, 0.05)	0.74	0.01 (−0.04, 0.05)	0.75
Bone density				
Bone mineral density (L1–L4, g/cm^2^)	−0.002 (−0.01, 0.003)	0.42	−0.001 (−0.01, 0.01)	0.77
T-score (L1–L4, g/cm^2^)	−0.02 (−0.06, 0.03)	0.45	−0.01 (−0.05, 0.04)	0.82
Z-score (L1–L4, g/cm^2^)	−0.02 (−0.06, 0.03)	0.49	−0.01 (−0.06, 0.04)	0.73

Breastfeeding is calculated as duration (months). Data are expressed as β (95% CI). Data marked by * were log-transformed.

*Adjusted for group allocation.

†Adjusted for group allocation, pre-pregnancy BMI, educational level, and glucose-lowering treatment in pregnancy.

AUC, area under the curve insulin/glucose; BIA, bioelectrical impedance analysis; BMI, body mass index; DEXA, dual-energy X-ray absorptiometry; GDM, gestational diabetes mellitus; HOMA-B, Homeostatic Model Assessment of β-cell index; HOMA-IR, Homeostatic Model Assessment for Insulin Resistance; IL-6, interleukin 6; ISSI-2, Insulin Secretion-Sensitivity Index-2; TNF-α, tumor necrosis factor α; Δ, change or difference.

Specifically, each supplementary month of breastfeeding was associated with 0.54 kg lower weight, 0.36 kg lower weight retention, 0.42–0.43 kg lower body fat (BIA or DXA), 0.26 kg lower fat-free mass (BIA), 0.22 kg lower lean mass (DXA), 0.11 kg/m^2^ lower FMI, and 0.01 kg lower VAT at 1 year postpartum, independent of group allocation, pre-pregnancy BMI, educational level, or need for glucose-lowering treatment during pregnancy. Breastfeeding duration was also inversely correlated with changes in weight, body fat (BIA), and fat-free mass (BIA) between 6–8 weeks and 1 year postpartum (all p≤0.05). The correlations between breastfeeding duration and the aforementioned outcome measures varied in strength, ranging from r=−0.16 for changes in fat-free mass in the postpartum (BIA) to r=−0.34 for body fat (DXA; data not shown).

Regarding insulin resistance and secretion, breastfeeding duration was negatively associated with HOMA-IR, HOMA-B and AUC (p<0.01), but positively associated with Matsuda and the insulin resistance-adjusted insulin secretion (ISSI-2) (all p≤0.01) at 1 year postpartum in model 1. The relationship between breastfeeding duration with HOMA-IR and HOMA-B was independent of the above-mentioned confounders (model 2; p≤0.02).

Regarding inflammation, we observed a significant negative correlation between breastfeeding duration and CRP (p<0.01) at 1 year postpartum that was independent of tested confounders (p=0.04). Breastfeeding duration was not associated with either TNF-α or IL-6, neither with BMD, T-score, or Z-score of the L1–L4 vertebrae at 1 year postpartum.

### Differences in metabolic health, inflammatory markers, and bone density according to breastfeeding category

Table 3 shows the differences and changes in metabolic health variables, inflammatory markers, and bone density at 1 year postpartum according to breastfeeding category.

**Table 3:** Differences and changes in metabolic health, C reactive protein (CRP), and bone density at 1 year postpartum according to breastfeeding category in women with prior GDM

n=171	BF <6 (n=69)	BF ≥6 (n=102)	Model 1*		Model 2†	
	**Mean±SD**	**Mean±SD**	**β (95% CI**)	**P value**	**β (95% CI**)	**P value**
Body composition						
Weight (kg)	78.6±17.9	68.5±14.0	−10.15 (−15.09, 5.21)	<0.001	−3.44 (−6.08, −0.79)	0.01
Weight retention (kg)	5.4±6.9	2.4±4.5	−2.92 (−4.66, 1.17)	<0.01	−2.40 (−4.24, 0.56)	0.01
Body fat (BIA, kg)	30.8±11.6	23.6±8.9	−7.11 (−10.29, 3.92)	<0.001	−2.72 (−4.47, 0.98)	<0.01
Fat-free mass (BIA, kg)	47.9±7.2	44.1±5.4	−3.77 (−5.73, 1.82)	<0.001	−1.55 (−2.93, 0.16)	0.03
ΔBody composition between 1 year and 6–8 weeks postpartum						
Weight difference (kg)	0.6±5.0	−1.7±4.3	−2.26 (−3.72, 0.80)	<0.01	−1.57 (−3.07, 0.07)	0.04
Body fat difference (BIA, kg)	−0.2±4.3	−2.3±3.7	−2.09 (−3.34, 0.84)	<0.01	−1.44 (−2.71, 0.18)	0.03
Fat-free mass difference (BIA, kg)	0.7±2.4	0.1±2.5	−0.68 (−1.45, 0.08)	0.08	−0.55 (−1.36, 0.26)	0.18
Body composition by DEXA						
Body fat (kg)	31.5±12.4	25.1±10.0	−6.43 (−10.93, 1.93)	0.01	−2.46 (−4.83, 0.09)	0.04
Lean mass (kg)	43.6±6.4	40.2±5.4	−2.82 (−5.19, 0.46)	0.02	−1.61 (−3.48, 0.26)	0.09
Fat mass index (kg/m^2^)	11.5±4.5	9.4±3.7	−2.09 (−3.75, 0.43)	0.01	−0.64 (−1.43, 0.15)	0.11
Visceral adipose tissue (kg)	0.7±0.5	0.5±0.4	−0.22 (−0.40, 0.03)	0.02	−0.07 (−0.17, 0.04)	0.20
Insulin resistance/secretion						
HOMA-IR	3.9±2.5	2.7±1.9	−1.25 (−1.94, 0.56)	<0.001	−0.57 (−1.15, 0.01)	0.06
HOMA-B	54.4±35.7	37.6±25.6	−16.82 (−26.38, 7.26)	<0.01	−7.38 (−15.66, 0.89)	0.08
Matsuda	4.8±3.7	6.0±3.4	1.22 (0.05, 2.39)	0.04	−0.14 (−1.13, 0.84)	0.77
AUC	0.6±0.5	0.5±0.2	−0.15 (−0.27, 0.04)	0.01	−0.06 (−0.17, 0.05)	0.27
ISSI-2	2.1±0.8	2.3±0.9	0.25 (−0.03, 0.53)	0.08	0.05 (−0.23, 0.33)	0.72
Inflammation						
CRP (mg/L)*	0.7 (−0.1, 1.6)	0.2 (−0.5, 0.9)	−0.52 (−0.88, 0.15)	0.01	−0.37 (−0.73, 0.01)	0.04
TNF-α (pg/mL)*	−0.6 (0.9, –0.3)	−0.7 (−1.1, –0.3)	−0.10 (−0.35, 0.15)	0.42	−0.08 (−0.33, 0.17)	0.53
IL-6 (pg/mL)*	−0.5 (−1.1, 0.1)	−0.7 (−1.1, 0.1)	−0.21 (−0.57, 0.15)	0.25	−0.23 (−0.61, 0.16)	0.25
Bone density						
Bone mineral density (L1–L4, g/cm^2^)	1.176±0.096	1.169±0.147	−0.01 (−0.06, 0.04)	0.79	0.01 (−0.05, 0.06)	0.81
T-score (L1–L4, g/cm^2^)	−0.1±0.8	−0.1±1.2	−0.05 (−0.48, 0.38)	0.82	0.06 (−0.38, 0.50)	0.79
Z-score (L1–L4, g/cm^2^)	−0.1±0.8	−0.2±1.2	−0.10 (−0.53, 0.33)	0.65	−0.02 (−0.47, 0.42)	0.91

Data are expressed as mean±SD and β (95% CI). Data marked by * were log-transformed and are expressed as median (IQR).

*Adjusted for group allocation.

†Adjusted for group allocation, pre-pregnancy BMI, educational level, and glucose-lowering treatment in pregnancy.

AUC, area under the curve insulin/glucose; BF <6, no or <6 months of breastfeeding; BF ≥6, ≥6 months of breastfeeding; BIA, bioelectrical impedance analysis; BMI, body mass index; DEXA, dual-energy X-ray absorptiometry; GDM, gestational diabetes mellitus; HOMA-B, Homeostatic Model Assessment of β-cell index; HOMA-IR, Homeostatic Model Assessment for Insulin Resistance; IL-6, interleukin 6; ISSI-2, Insulin Secretion-Sensitivity Index-2; TNF-α, tumor necrosis factor α; Δ, change or difference.

Compared with BF <6 women, BF ≥6 women had significantly lower weight, weight retention, body fat (BIA and DXA), and fat-free mass (BIA) (all p≤0.04) at 1 year postpartum independent of confounders (model 2). Notably, the weight of BF ≥6 women was 3.44 kg lower, weight retention was 2.40 kg lower, and body fat (BIA) was 2.72 kg lower compared with BF <6 women. In addition, BF ≥6 women had greater weight and body fat (BIA) loss between 6–8 weeks and 1 year (all p≤0.04). BF <6 women had increased insulin resistance (higher HOMA-IR and lower Matsuda) and higher insulin secretion (HOMA-B and AUCins/glu) compared with BF ≥6 women (all p≤0.04) at 1 year postpartum; however, these associations were not significant when we adjusted for confounders.

Compared with BF <6 women, BF ≥6 women had significantly lower CRP at 1 year postpartum, which was independent of confounders (p=0.046). There was no significant difference in TNF-α, IL-6, BMD, T-score, or Z-score between both breastfeeding groups.

## Discussion

In this multiethnic cohort of women with GDM, we prospectively investigated associations between breastfeeding and metabolic health, inflammation and bone density in the postpartum period. We observed that a longer duration of breastfeeding was associated with lower weight, weight retention, body fat, and VAT at 1 year postpartum. It was also associated with decreased fat-free mass, although to a lower extent. Despite this, it had no unfavorable impact on BMD. Breastfeeding duration was also related to lower CRP, and lower measures of insulin resistance (HOMA-IR) and insulin secretion (HOMA-B, AUCins/glu), as well as to increased overall insulin sensitivity (Matsuda) and insulin resistance-adjusted insulin secretion (ISSI-2). All the observed associations, except AUCins/glu, Matsuda, and ISSI-2, were independent of medical and sociodemographic confounder variables. Similar results were found when we compared breastfeeding for ≥6 months, as encouraged by international recommendations, to a shorter breastfeeding duration.

Associations between breastfeeding duration and lower weight, weight retention, body fat, and FMI during the postpartum are consistent with a previous meta-analysis showing that a longer breastfeeding duration of any intensity is associated with lower BMI among women with prior GDM,^7^ but is in contrast to more recent studies.^20 25^

In our study, breastfeeding duration was inversely associated with VAT. Even in the absence of PPWR, VAT has been shown to remain higher than before pregnancy^54^ and is associated with an increased risk of cardiometabolic diseases.^55^ Our results are in agreement with previous studies in women without GDM,^17 18^ but not with two studies in women after GDM.^20 21^ The small sample size (n=26), early evaluation after only 3 months,^21^ as well as the differences in GDM criteria resulting in a population with an advanced glucose intolerance in these studies^20 21^ might explain the differences.

Variations in study designs, dietary advice, and the cultural and ethnic backgrounds of participants can influence differences in weight loss after GDM.^20 25^ The beliefs and practices regarding breastfeeding and concomitant or compensatory increases in food intake in this period may vary significantly. Our multiethnic participants received consistent dietary messages advising against substantial increases in food intake during breastfeeding.^35^ These lifestyle adaptions together with longer breastfeeding might account for the pronounced effect of breastfeeding on weight and body composition observed in our cohort compared with other studies. For instance, women in our cohort displayed 0.36 kg lower weight per month of breastfeeding, compared with two studies that reported a weight retention reduction equivalent to 0.04–0.17 kg per month in the general population.^56 57^

Our finding of an association between breastfeeding and decreased lean mass and fat-free mass is in contrast to a prospective study in women without GDM, which found no correlation,^11^ but noted that breastfeeding women in their cohort had a high protein intake. Lean mass is associated with many health benefits, including increased metabolic rate, reduction in inflammatory markers, and increased BMD.^10 58^ In our cohort, we found no relationship between breastfeeding and BMD at 1 year postpartum. Breastfeeding has been shown to reduce BMD, particularly within the first 6 months postpartum, but most studies show a full bone mass recovery afterwards.^59^ Postulated reasons for this temporary loss are mainly calcium mobilization from the bones used for milk production, higher parathyroid hormone-related protein stimulating the release of calcium from the bones, lower calcium intestinal absorption and high prolactin levels suppressing the hypothalamic–pituitary–ovarian axis.^13 59^ From our results, it appears that breastfeeding duration in women with GDM does not negatively impact BMD at 1 year postpartum (even in a context where 28% of women were still breastfeeding), aligning with results in women without GDM. These results are reassuring, especially since women with prior GDM have lower BMD^30^ compared with healthy controls and have an increased risk of hip and other fractures later in life.^31^ The long-term effects of breastfeeding on BMD in women with previous GDM warrant further investigation.

Breastfeeding duration was associated with lower insulin resistance (HOMA-IR) and lower insulin secretion (HOMA-B). Regarding HOMA-IR, our results are consistent with previous studies in women with GDM.^7 25^ Other studies in this population have not shown an association between breastfeeding and insulin secretion such as HOMA-B,^20 25^ although correlation was close to significance in one of them.^25^ Unadjusted results in our cohort also showed an association between breastfeeding and the insulin resistance-adjusted secretion, the ISSI-2, consistent with two previous studies. These studies did not adjust for potential confounders,^21 22^ and in our analysis, the association between breastfeeding and ISSI-2 was attenuated after adjusting for pre-pregnancy BMI. These results suggest that breastfeeding mainly improves insulin resistance but its impact on insulin secretion might be mediated through the effect on weight and potentially insulin resistance. Indeed, when we adjusted for HOMA-IR, the association between breastfeeding and ISSI-2, as well as HOMA-B, was not significant (data not shown).

In our cohort, breastfeeding duration, as well as breastfeeding for ≥6 months, was associated with decreased CRP, a marker of chronic low-grade inflammation. This association also persisted after adjustments for confounders, including pre-pregnancy BMI. These findings might further help to explain the relationship between breastfeeding and less cardiometabolic outcomes, as low-grade inflammation predicts increased adverse metabolic risk.^60^ Previous research has shown mixed results regarding longer breastfeeding duration and lower postpartum CRP.^20 23 25 26^ One study found a significant association between breastfeeding duration and lower CRP in middle-aged women with a history of GDM.^26^ It is worthy to point out that in our cohort, breastfeeding was not associated with TNF-α or IL-6 at 1 year postpartum. Different mechanisms might be involved in the long-term and short-term regulation and expression of these inflammatory markers in the blood in women with GDM, suggesting that the impact of breastfeeding might not be the same.

Our study has several strengths. We prospectively followed women with GDM during pregnancy up to 1 year postpartum. To our knowledge, this is the first study to investigate the relationship between breastfeeding duration with diverse health outcomes including body fat, lean mass, BMD, and inflammatory markers at 1 year postpartum in women with prior GDM. Furthermore, we evaluated an exhaustive number of metabolic markers, to assess the overall effect of breastfeeding on metabolic health in women with prior GDM and included also bone health in the same population. Many potential confounding variables including adverse pregnancy outcomes and lifestyle parameters in the postpartum were investigated. Moreover, our study is the first to illustrate the beneficial effect of breastfeeding ≥6 months compared with <6 months in women with prior GDM. Potential limitations in our study include the lack of a control group (women without GDM). In addition, the lack of data on the frequency and exclusivity of breastfeeding is a limitation, considering the dose–response relationship between breastfeeding intensity and metabolic health in women.^16 61 62^ Furthermore, 40 participants had missing data on breastfeeding duration (n=40) which could influence our results, as they had a higher pre-pregnancy BMI and HbA1c levels at the first GDM visit. As previous studies show the association between higher maternal weight and lower rates of breastfeeding initiation and shorter breastfeeding duration,^63^ it is possible that patients with missing breastfeeding duration data would have had a lower mean breastfeeding duration. Finally, we did not account for potential confounders affecting BMD, such as vitamin D or calcium intake, which may limit some of the interpretability of our findings.

## Conclusions

In this multiethnic prospective cohort of women with GDM followed during pregnancy up to 1 year postpartum, longer breastfeeding duration was associated with lower metabolic parameters including weight, weight retention, body fat, VAT, CRP, and lower insulin resistance at 1 year postpartum, independent of confounders. Each month of breastfeeding duration was associated with a 0.54 kg lower weight and 0.43 kg lower body fat. Our findings suggest that breastfeeding for ≥6 months is protective of these adverse metabolic outcomes. In addition, breastfeeding ≥6 months is desirable, not only for the infants, but also for the cardiometabolic health of these high-risk mothers with no observed adverse effect on BMD. Therefore, we suggest integrating breastfeeding for at least 6 months in clinical care recommendations for women after GDM. Healthcare professionals should emphasize the potential enhancement of the benefits of breastfeeding through concurrent healthy diet and lifestyle choices when advising women on breastfeeding.

## Data Availability

Data are available upon reasonable request. Not applicable.
