# Supplementary material for: Prospective associations between breast feeding, metabolic health, inflammation and bone density in women with prior gestational diabetes mellitus
Source: BMJ Open Diabetes Res Care. 2024 May 21;12(3):e004117. doi: 10.1136/bmjdrc-2024-004117 (PMC11110608; doi:10.1136/bmjdrc-2024-004117)
Supplement: Supplementary data [file bmjdrc-2024-004117supp001.pdf]

Supplementary Table 1 Adverse pregnancy outcomes according to breastfeeding category in women with prior GDM

	**All (n=171) n (%)**	**BF$^{<6}$ (n=69) n (%)**	**BF$^{\geq 6}$ (n=102) n (%)**	**p-value**
Pregnancy-induced hypertension (n=124)				0.23
Yes	2 (1.6)	0 (0.0)	2 (2.7)	
Preeclampsia during pregnancy (n=98)				0.76
Yes	3 (3.1)	1 (2.4)	2 (3.5)	
Placenta previa during pregnancy (n=92)				0.24
Yes	1 (1.1)	1 (2.6)	0 (0.0)	
Cesarean section (n=162)				0.17
Yes	61 (37.7)	29 (43.9)	32 (33.3)	
Prematurity (n=169)				0.60
Yes	15 (8.9)	7 (10.3)	8 (7.9)	
Intrauterine growth restriction (n=170)				0.90
Yes	7 (4.1)	3 (4.4)	4 (4.0)	
Small for gestational age (n=169)				0.10
Yes	21 (12.4)	12 (17.4)	9 (9.0)	
Large for gestational age (n=169)				0.38
Yes	19 (11.2)	6 (8.7)	13 (13.0)	
Hospitalization in neonatology (n=171)				0.87
Yes	8 (4.7)	3 (4.4)	5 (4.9)	

Data are expressed as n (%). BF$^{<6}$ denotes no or <6 months of breastfeeding, BF$^{\geq 6}$ denotes ≥6 months of breastfeeding.
