# Supplementary material for: Prospective associations between breast feeding, metabolic health, inflammation and bone density in women with prior gestational diabetes mellitus
Source: BMJ Open Diabetes Res Care. 2024 May 21;12(3):e004117. doi: 10.1136/bmjdrc-2024-004117 (PMC11110608; doi:10.1136/bmjdrc-2024-004117)
Supplement: Supplementary data [file bmjdrc-2024-004117supp002.pdf]

Supplementary Table 2 Differences in lifestyle factors at 1-year postpartum according to breastfeeding category in women with prior GDM

	**BF$^{<6}$ (n=69)**	**BF$^{\geq 6}$ (n=102)**	**Model 1[a]**		**Model 2[b]**	
**Lifestyle factors**	**mean±SD**	**mean±SD**	**β (95%CI)**	**p-value**	**β (95%CI)**	**p-value**
Food intake (daily total calorie consumption, kcal/day) (n=160)	1575.3±1250.3	1518.7± 735.4	-51.62 (-363.23, 260.00)	0.74	-49.62 (-380.38, 282.14)	0.77
Daily total physical activity (min/day) (n=119)	309.8±79.0	322.2±96.6	14.61 (-18.72, 47.95)	0.39	14.57 (-20.60, 49.73)	0.41
Time spent in moderate and vigorous physical activity (min/day) (n=119)	144.3± 60.5	148.2± 66.2	4.90 (-18.95, 28.75)	0.69	3.12 (-21.77, 28.00)	0.80

Data are expressed as mean±standard deviation and beta (95% confidence interval). BF$^{<6}$ denotes no or <6 months of breastfeeding; BF$^{\geq 6}$ denotes ≥6 months of breastfeeding.

[a] Adjusted for group allocation.

[b] Adjusted for group allocation, pre-pregnancy BMI, educational level and glucose-lowering treatment in pregnancy.
